# GEANT4-DNA simulation of temperature-dependent and pH-dependent yields of chemical radiolytic species

**DOI:** 10.1088/1361-6560/acd90d

**Published:** 2023-06-15

**Authors:** Jingyi Bian, Juan Duran, Wook-Geun Shin, Jose Ramos-Méndez, Jack C Sankey, Lilian Childress, Jan Seuntjens, Shirin A Enger

**Affiliations:** 1 Medical Physics Unit, Department of Oncology, Faculty of Medicine, McGill University, Montreal, Quebec, Canada; 2 Physics Division, Department of Radiation Oncology, Massachusetts General Hospital & Harvard Medical School, Boston, MA-02114, United States of America; 3 Department of Radiation Oncology, University of California San Francisco, CA, United States of America; 4 Department of Physics, McGill University, Montreal, Quebec, Canada; 5 Research Institute of the McGill University Health Centre, Montreal, Quebec, Canada; 6 Lady Davis Institute for Medical Research, Jewish General Hospital, Montreal, Quebec, Canada

**Keywords:** radiation chemical yield, water radiolysis, radiation chemistry, GEANT4-DNA

## Abstract

**Objective.:**

GEANT4-DNA can simulate radiation chemical yield (*G*-value) for radiolytic species such as the hydrated electron $\left({\text{e}}_{\text{aq}}^{-}\right)$ with the independent reaction times (IRT) method, however, only at room temperature and neutral pH. This work aims to modify the GEANT4-DNA source code to enable the calculation of *G*-values for radiolytic species at different temperatures and pH values.

**Approach.:**

In the GEANT4-DNA source code, values of chemical parameters such as reaction rate constant, diffusion coefficient, Onsager radius, and water density were replaced by corresponding temperature-dependent polynomials. The initial concentration of hydrogen ion $\left({\text{H}}^{+}\right)$/hydronium ion $\left({\text{H}}_{3}{\text{O}}^{+}\right)$ was scaled for a desired pH using the relationship $\text{pH}=-{\text{log}}_{10}\left[{\text{H}}^{+}\right]$. To validate our modifications, two sets of simulations were performed. (A) A water cube with 1.0 km sides and a pH of 7 was irradiated with an isotropic electron source of 1 MeV. The end time was 1 μs. The temperatures varied from 25 °C to 150 °C. (B) The same setup as (A) was used, however, the temperature was set to 25 °C while the pH varied from 5 to 9. The results were compared with published experimental and simulated work.

**Main results.:**

The IRT method in GEANT4-DNA was successfully modified tosimulate *G*-values for radiolytic species at different temperatures and pH values. Our temperature-dependent results agreed with experimental data within 0.64%–9.79%, and with simulated data within 3.52%–12.47%. The pH-dependent results agreed well with experimental data within 0.52% to 3.19% except at a pH of 5 (15.99%) and with simulated data within 4.40%–5.53%. The uncertainties were below ±0.20%. Overall our results agreed better with experimental than simulation data.

**Significance.:**

Modifications in the GEANT4-DNA code enabled the calculation of *G*-values for radiolytic species at different temperatures and pH values.

## 1. Introduction

### 1.1. Water Radiolysis

When ionizing radiation with sufficient energy interacts with water, it deposits energy along particle tracks, decomposing the water molecules and forming clusters of reactive species known as spurs at each energy deposition point. This process is called water radiolysis (Buxton et al 1988, Spinks and Woods 1990). Inside the spurs, there exists a competition between the diffusion and reaction of these species as the non-homogeneous concentration gradients relax. Reaction (1) presents a list of the primary and secondary species created in the water radiolysis. The primary species comprise the $e_{aq}^{-}$, OH•, ${\text{H}}_{\text{3}}{\text{O}}^{\text{+}}$, ${\text{OH}}^{-}$, ${\text{H}}^{\text{+}}$^,^ and ${\text{H}}_{\text{2}}$. While the secondary species encompass the ${\text{H}}^{•}$, ${\text{H}}_{2}{\text{O}}_{2}$, and ${\text{H}}_{2}$. It is noteworthy that ${\text{H}}_{\text{2}}$ is primarily generated during the initial act of water radiolysis rather than through intraspur reactions, which classifies it as both a primary species and a secondary species (Bui et al 2023). Radiolysis of water is divided into three stages: physical stage, physico-chemical stage, and chemical stage (Draganicé and Draganicé 1971, Spinks and Woods 1990). The physical stage starts with the initial energy deposition in water leading to the formation of ionized water molecules $\left({\text{H}}_{2}{\text{O}}^{+}\right)$, excited water molecules $\left({\text{H}}_{2}{\text{O}}^{*}\right)$, and sub-excitation electrons $\left({\text{e}}_{\text{sub}}^{-}\right)$. The physical stage lasts up to 10^−15^ s after the initial interaction with ionizing radiation. The physico-chemical stage follows from 10^−15^ to 10^−12^ s and consists of processes including ion-molecule reactions, dissociative relaxation, and thermalization (solvation) of sub-excitation electrons (Draganicé and Draganicé 1971).

(1) H2O→[IR]eaq−,H•,OH•,H3O+,H2O2,H2,H+

The chemical stage is the final stage and takes place from 10^−12^ to 10^−6^ s. During this stage, the species in the spurs undergo intraspur reactions while others diffuse away from the original point. By 10^−6^ s, a homogeneous distribution of the species is assumed (Draganicé and Draganicé 1971, Le Caër 2011). The $e_{aq}^{-}$ is a highly reactive and short-lived species. The recombination reactions of $e_{aq}^{-}$ occur in the chemical stage (Walker 1967). Moreover self-reactions of OH• and H^•^ also occur in this stage (Hervé du Penhoat et al 2000). The ${\text{H}}^{•}$ reacts with water.

### 1.2. Radiation chemical yield

The *G*-value, defined as the number of chemical species created or lost per 100 eV of energy deposited, was introduced in the 1940s by Burton (1947). Obtaining accurate *G*-values is important in many domains, including the modeling of DNA damage (Villagrasa et al 2017, Lampe et al 2018, Sakata et al 2019) and radiation dosimetry (Schuler and Allen 1956, Hart and Boag 1962, Judeikis et al 1968, Hart and Fielden 1970, Mégrourèche 2020). The *G*-values for different primary species formed in the water radiolysis are dependent on many physical parameters such as the linear energy transfer (LET) of the incoming radiation, temperature, and pH value of the irradiated solution (Draganicé and Draganicé 1971, Autsavapromporn et al 2007, Plante 2011b).

It is difficult to measure the concentration of the reactive species directly (Draganicé and Draganicé 1971). First, under normal experimental conditions, the reactive species’ concentration is very low (fractions of a micromole), which requires highly sensitive equipment. The sensitivity of the experimental equipment can significantly increase the uncertainty of the results. Second, the species’ lifetime is short (microsecond), which requires equipment capable of performing rapid measurements. Third, during the measurements, it is necessary to wait until a minimum measurable concentration is reached. The accumulation of products often complicates the observed phenomenon, as the products being accumulated may themselves be susceptible to radiation-chemical changes (e.g. ${\text{e}}_{\text{aq}}^{-}$ can react with itself). Finally, measurement of the *G*-values for radical species is often based on a product analysis, which involves adding certain solutes before irradiation. To determine the *G*-values, chemical changes of the solutes are monitored while they react with the species under investigation. However, it is difficult to find solutes that are soluble in water and do not affect its pH (Draganicé and Draganicé 1971, Yamashita et al 2008). Overall, the direct measurement of *G*-values is challenging. Due to these limitations, simulations with computational models including the Monte Carlo track structure codes were proposed (Uehara and Nikjoo 2006). Several studies have used Monte Carlo track structure simulations to investigate different factors such as the energy of the incident radiation (LaVerne et al 2005, Bui et al 2023), cluster size (Rudek et al 2019), and LET (Plante 2011b) affecting the *G*-values. Monte Carlo simulations also have been used to investigate dependencies on temperatures and pH values (Hervé du Penhoat et al 2000, Autsavapromporn et al 2007, Plante 2011b, Sanguanmith et al 2012).

### 1.3. GEANT4-DNA

Many Monte Carlo track structure codes have been developed to simulate event-by-event radiation interaction in water and the formation of radiolytic species (Uehara et al 2001, Valota et al 2003, Nikjoo et al 2006, Kreipl et al 2009, Grosswendt et al 2014,* Boscolo et al 2018) including the GEANT4-DNA package (Incerti et al 2010a, 2010b, Bernal et al 2015, Incerti et al 2018), which extends on the open-source GEANT4 Monte Carlo simulation toolkit (Incerti et al 2010b). However, since GEANT4-DNA is a track structure code, all the ionizations along the charged particle tracks through water are simulated, making the code computationally expensive. In addition to this, the simulation of water radiolysis conventionally is based on a step-by-step (SBS) approach adding to the computational cost both in power and time (Kreipl et al 2009, Incerti et al 2010b, Plante 2011a, Karamitros et al 2014, Shin et al 2019). To alleviate this, variance reduction techniques (Ramos-Méndez et al 2018) and a combination of condensed-history and track-structure transport (Ivanchenko et al 2011) were implemented during the physical stage while the independent reaction times (IRT) technique was implemented for simulating the reaction kinetics of chemical species during the chemical stage (Ramos-Méndez et al 2020).

The IRT method is well suited for water radiolysis simulations due to its efficiency compared with the SBS method. As of today, the simulation of water radiolysis with varying temperatures and pH values using GEANT4-DNA has not been reported. Simulation of water radiolysis with varying temperatures and pH values, and studying the influence of these parameters on the *G*-values for the generated radical species need to be further investigated.

## 2. Aim

The aim of this study was to further develop the GEANT4-DNA source code to allow users to obtain *G*-values for reactive species produced in water radiolysis at different temperatures and pH values. This study also aims to perform simulations of *G*-values for these species to validate the modifications in the code.

### 3. Materials and methods

In the following section, a brief summary of the implementation of the IRT method in GEANT4-DNA is given, which is necessary to understand the changes made in the code for obtaining the *G*-values for reactive species at different temperatures and pH values.

#### 3.1. Chemical parameters in water radiolysis simulation

Chemical parameters that have an impact on the simulation process of water radiolysis in the IRT method are the reaction rate constant, diffusion coefficient, the Onsager radius $\left(r_{c}\right)$, and water density (Clifford et al 1982a, 1982b, 1986, Goulet et al 1998, Plante 2011a, Shin et al 2019). Reaction rate constants are used to quantify the rate and direction of the water radiolysis reactions and have a great impact on the water radiolysis process. The diffusion coefficients are used to describe the diffusional motion of molecules in solution or the kinetics of reactions between reactants. The Smoluchowski diffusion equation and the Debye-Smoluchowski equation have been widely used to simulate this transportation. A detailed derivation of the theory of diffusion is presented in published work (Clifford et al 1984, Green et al 1989, Berg 1993, Karamitros et al 2014).

The reaction radius, R, which refers to the threshold at which the reactants can react is calculated by the Smoluchowski diffusion equation:

(2) $$R=\frac{k}{4\pi N_{\text{A}}D},$$

where $N_{A}$ is Avogadro’s number, k is the effective reaction rate constant (including re-dissociation), and D is the sum of the diffusion coefficients of molecules. The radical species are diffused based on their temperature-dependent coefficients. In general, an electron is simulated down to the energy limit of the physical models, then it is stopped and moved a distance randomly sampled from an energy-dependent thermalization curve (Mozumder 1999, Meesungnoen et al 2002). Hervé du Penhoat et al (2000) studied the effect of temperature on this thermalization distance and found that the electron thermalization distance decreases with increasing temperature. The Onsager radius represents the range of the Coulomb interaction in a particular system. It is defined as the distance at which the electrostatic energy of a pair of elementary charges (electrical charge $e_{A}$ and $e_{B}$) falls to the thermal level. The probability of reaction for diffusion-controlled reactions between charged particles is affected by the Onsager radius (Green et al 1989, 1990, Plante 2011a). The temperature-dependent Onsager radius is defined by the equation (3):

(3) $$r_{c}=\frac{e_{A}e_{B}}{4\pi \varepsilon k_{B}T},$$

where $k_{B}$ is Boltzmannʼs constant, ε is the relative permittivity of the solvent (water), and T is the absolute temperature of the medium in Kelvin (for water, $r_{c}\approx 0.715$ nm at 25 °C ) from (Karamitros et al 2014).

The IRT method takes information from a particle’s position at the end of the pre-chemical stage, as well as parameters such as the reaction rate constants and diffusion coefficients, to calculate the reaction time of a given reaction (Sano and Tachiya 1979, Clifford et al 1984). The water radiolysis simulations with the GEANT4-DNA using the IRT method result in a large number of reactions, e.g. 15 species and 72 reactions for the time beyond the microsecond range that includes both heterogeneous and homogeneous chemistry stages (Elliot and McCracken 1990, Pastina and LaVerne 2001). For the time range below 1 μs, previous studies demonstrate that between 10 and 14 reactions are sufficient to obtain accurate *G*-values compared to measured data (Hervé du Penhoat et al 2000, Ramos-Méndez et al 2022). The reaction rate constants are presented in Tables A1–A8 in Appendix A, and the diffusion coefficients are presented in tables B1 and B2 in Appendix B. As presented in the Appendices, chemical parameters, such as reaction rate constant and diffusion coefficient, can be described as functions of temperature. These parameters were made temperature-dependent in this study, by taking values from the literature (Burns and Marsh 1981, Elliot 1994, Schmidt et al 1995, Elliot and Bartels 2009, Kallikragas et al 2014).

The density of water also changes with temperature along the liquid–vapour coexistence curve (Irvine and Hartnett 1976), as shown in equation (4). That is, there exist pressure-temperature combinations at which the two phases can coexist, as described by the liquid–vapor coexistence curve (de Pablo et al 1990, Bauer and Patel 2009)

(4) $$\rho \left(\text{g}\;{\text{ml}}^{-1}\right)=0.999+1.094\times 10^{-4}t-7.397\times 10^{-6}t^{2}+2.693\times 10^{-8}t^{-3}-4.714\times 10^{-11}t^{4}.$$

### 3.2. Simulation Setup

In this project, additional flexibility was added to GEANT4-DNA to configure the input chemical parameters of the IRT method. The GEANT4-DNA package version 10.07 was used. The values of the chemical parameters mentioned above (reaction rate constant, diffusion coefficient, the Onsager radius, and water density) at ambient temperature (25 °C) were replaced with corresponding temperature-dependent polynomials. Reaction rate constants presented in tables A1–A8 in Appendix A, were made temperature-dependent by taking the values from (Burns and Marsh 1981, Elliot 1994, Elliot and Bartels 2009). Diffusion coefficients presented in tables B1, and B2 were made temperature-dependent by taking the values from published work (Elliot 1994, Schmidt et al 1995, Kallikragas et al 2014). The temperature-dependent Onsager radius described in equation (3) decreases as the temperature increases from 25 °C to 150 °C. As shown in equation (4), all simulations were performed with the liquid–vapor coexistence curve. As the temperature increases from 25 °C to 150 °C, the density of pressurized water varies from 1 g ml^−1^ (0.003 MPa) to 0.917 g ml^−1^ (0.477 MPa) (Linstorm 1998).

Regarding the pH dependence, pH generally represents the concentration of ${\text{H}}_{3}{\text{O}}^{+}$ and ${\text{OH}}^{-}$, which affects the type 6 reaction rates as described in section A and presented in table A8. Since one of the reactants in type 6 reactions has a considerably bigger concentration than other reactants and is considered a background molecule, the reaction rate is the product of the observed reaction rate of the reaction and the concentration of the background reactant. The product is called the scavenger capacity and is considered to be the reaction rate of the reaction (Plante and Devroye 2017). Background molecules in this model are the ${\text{H}}_{3}{\text{O}}^{+}$, the ${\text{OH}}^{-}$, and water (${\text{H}}_{2}\text{O}$). The concentration of ${\text{OH}}^{-}$ and ${\text{H}}_{3}{\text{O}}^{+}$ varies depending on the input pH.

In addition, several changes were made to the chemistry modules of the GEANT4-DNA source code. Two new methods for enabling change of the temperature and the pH by the users were added to the G4DNAChemistryManager class (G4DNAChemistryManager Class Reference, n.d.). This class is called from the physics models and is responsible for creating the water molecules and the solvated electrons and sending them to the G4ITStepManager class to be treated in the chemistry stage. A new constructor that takes temperature and pH as input was added to the G4EmDNAChemistry _ option3 class (Cobut et al 1998, Shin et al 2021), which defines molecules, chemical reactions, and dissociation schemes (Shin 2020). All molecules’ temperature-dependent diffusion coefficient values (as presented in tables B1, and B2) were instantiated in this class. A new method called ConstructReactiontablePhTemp was implemented to initialize the reaction rate constants of all the reactions with temperature-dependent polynomials (presented in tables A1–A8), and the concentrations of ${\text{OH}}^{-}$ and ${\text{H}}_{3}{\text{O}}^{+}$, which vary depending on the input pH. In addition, the G4DNAMolecularReactiontable class, which contains a table of chemical reactions and parameters (Shin 2020), was modified so that the users can set and get the temperature for the solution. The Onsager radius with temperature-dependent polynomial was initialized in this class. Apart from the scaling of temperature and pH-dependent values, all algorithms, chemical and physical models remained unchanged. G4EmDNAPhysics _ option2 (Champion et al 2009, Incerti et al 2018) physics constructor was used in this work.

To enable GEANT4-DNA users to benefit from the added features, a GEANT4-DNA example user code called chem6 was modified (Shin et al 2019). After the modifications, the user code was called Chem _ Temp _ pH. Water density with temperature-dependent polynomial was initialized in the DetectorConstruction class of Chem _ Temp _ pH. In addition, a constructor that takes the temperature and pH of the solution as input was added to its PhysicsList class.

### 3.3. Validation of the modification

Two sets of simulations were performed to validate our additions to the GEANT4-DNA source code. In the first set of simulations, *G*-values’ dependency on temperature (from 25 °C to 150 °C) for radiolytic species was examined. A semi-infinite water cube (mimic as 1 km sides) was irradiated with an isotropic point source of 1 MeV electrons placed in the center of the phantom. The rationale for using 1 MeV electrons was based on the upper limit for electron interaction cross-sections in GEANT4-DNA, and to simulate a setup that was closer to published pH and temperature studies, such that we could compare our results with these published studies. Experiments performed by Elliot et al (1993) are conducted with 2.25 MeV electrons. However, as described above, GEANT4-DNA has an upper energy limit of 1 MeV for electron interactions. Nevertheless, Pimblott and LaVerne (1998) demonstrate that above 100 keV, the *G*-values produced by electrons are unaffected by the electron’s initial kinetic energy. The 1 km side water cube was chosen to mimic an infinite volume to ensure that all the secondary particles and reactive species do not leave the volume (Bui et al 2023). Primary electrons with the incoming kinetic energy of 1MeV were killed if deposited energy was greater than 10 keV by the primary killer class, thus ensuring a constant ionization density (Pimblott and LaVerne 1998, Karamitros et al 2011). The end time for the simulation was set to 1 μs due to the homogeneous distribution of the radical and molecular products, which is assumed by about 1 μs after the ionizing event (end of the chemical stage). The water pH was set to 7. The simulations were performed at 25 °C, 50 °C, 75 °C, 100 °C, 125 °C and 150 °C. The second set of simulations examined the dependency of *G*-values on pH. The same phantom and source characteristics were used. However, the temperature was kept constant at a value of 25 °C, and the simulations were performed at pH values of 5, 6, 7, 8, and 9. Ten runs were performed, with each run consisting of 1000 incoming electrons. Each run took minutes.

In this work, data was collected from 10^−3^ to 1000 μs. Totally 50 data points were collected in this time range. *G*-values at 1 μs were collected and compared with published experimental and simulation data. Percentage differences between the results in this work and published data were calculated to verify our implementation of the GEANT4-DNA package. Another way of verifying our implementation was to perform the *material balance test*, which assumes a balance between the reducing species and oxidizing species produced in the water radiolysis process (Allen 1961, Draganicé and Draganicé 1971, Hervé du Penhoat et al 2000). The material balance test verifies if the chemical system conserves material by computing the equilibrium states which evolve from initial states through the processes of chemical reaction and diffusion (Edelen 1975). The material balance can be expressed as the equation below:

(5) $$G_{red}=G_{ox}$$

(6) $$G_{red}=G\left(e_{aq}^{-}\right)+2G\left({\text{H}}_{2}\right)+G\left({\text{H}}^{•}\right)$$

(7) Gox=G(OH•)+2G(H2O2)+3G(HO2)

## 4. Results and discussion

### 4.1. Effect of temperature on *G*-values for reactive species

**Figure 1.**
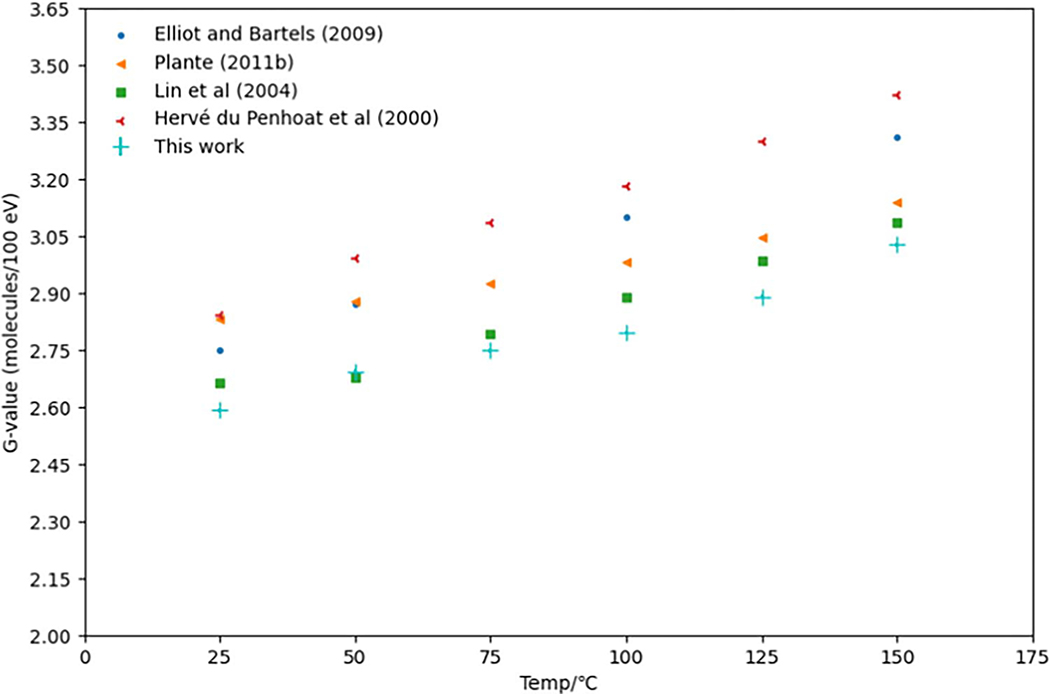
$\text{G}\left(e_{aq}^{-}\right)$ at the temperature range from 25 °C to 150 °C simulated in this work and comparison with the published work. The results were compared with the published experimental data (Lin et al 2004, Elliot and Bartels 2009), and simulation results from Monte Carto track structure codes IONLYS-TRACION and IONLYS-TRACELE (Plante 2011b) and from TRACIRT (Hervé du Penhoat et al 2000).

Figure 1 shows the $\text{G}\left(e_{aq}^{-}\right)$ at different temperatures from 25 °C to 150 °C simulated in this work. The results were compared with the published experimental data (Lin et al 2004, Elliot and Bartels 2009) and simulation results from Monte Carto track structure codes IONLYS-TRACION and IONLYS-TRACELE (Plante 2011b) and from TRACIRT (Hervé du Penhoat et al 2000). The $\text{G}\left(e_{aq}^{-}\right)$ at all temperature points shown were: 2.59 (25°C), 2.69 (50 °C), 2.75 (75 °C), 2.80 (100 °C), 2.89 (125 °C) and 3.03 (150 °C). The uncertainties were averaged across all simulated particles and then propagated across all executed runs. In this work, the uncertainties of the temperature-dependent $\text{G}\left(e_{aq}^{-}\right)$ were less than 0.18%. Our results agreed with the experimental data within 0.64 ± 0.18% to 9.79 ± 0.16% (Lin et al 2004, Elliot and Bartels 2009). Considering the difficulties of temperature and pressure control in the experimental setup and measurement errors in determining the radiolytic yields, the results are acceptable. Regarding simulation results performed with other code packages, our results agreed with the data within 3.52 ± 0.17% to 12.47 ± 0.15% (Hervé du Penhoat et al 2000, Plante 2011b). As can be seen in Figure 1, overall our results agreed better with published experimental work than with simulation results performed with other code packages. With increasing temperature, the $\text{G}\left(e_{aq}^{-}\right)$ undergoes a gradual increase primarily due to two processes: self-recombination of $e_{aq}^{-}$ and its reactions with other primary or secondary species. The recombination of $e_{aq}^{-}\left(e_{aq}^{-}+e_{aq}^{-}\to {\text{H}}_{2}+2{\text{OH}}^{-}\right)$ and its reaction with ${\text{H}}^{+}\left(e_{aq}^{-}+{\text{H}}^{+}\to {\text{H}}^{•}\right)$ are both controlled by diffusion, while its reaction with OH•(eaq−+OH•→OH−) is partially influenced by diffusion. Although the diffusion of species increases with temperature, the reaction rate constant of the reaction between $e_{aq}^{-}$ and OH• does not increase proportionally. As the temperature rises, a greater number of $e_{aq}^{-}$ become available for either diffusing out of the spur or participating in the spur’s reactions through self-recombination and reaction with ${\text{H}}^{•}$ to form various molecular products, predominantly ${\text{H}}_{2}$. Overall, both $\text{G}\left(e_{aq}^{-}\right)$ and $\text{G}\left({\text{H}}_{2}\right)$ demonstrate an upward trend with increasing temperature. (Elliot and Ouellette 1994, Elliot et al 1996, Hervé du Penhoat et al 2000).

**Figure 2.**
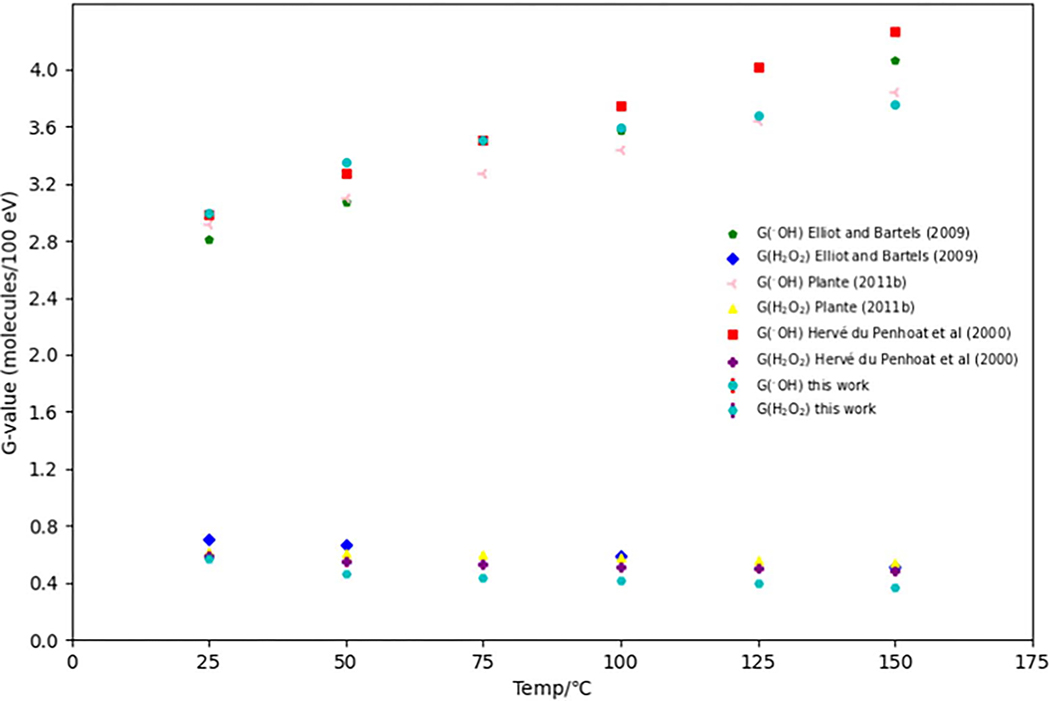
$\text{G}{(}^{•}\text{OH})$ and $\text{G}\left({\text{H}}_{2}{\text{O}}_{2}\right)$ were simulated in this study within the temperature range of 25 °C to 150 °C. These simulated values were then compared to published experimental data (Elliot and Bartels 2009), as well as simulation results obtained from Monte Carlo track structure codes IONLYS-TRACION and IONLYS-TRACELE (Plante 2011b), and TRACIRT(Hervé du Penhoat et al 2000).

**Figure 3.**
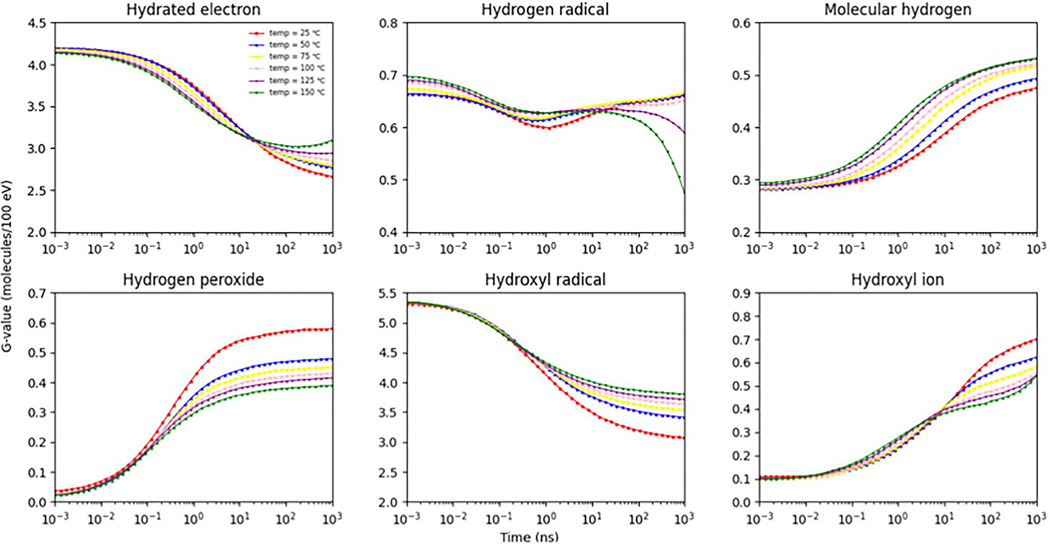
Time-evolution of *G*-values for generated reactive species at different temperatures from 25 °C to 150 °C for incoming electron energy of 1 MeVand pH value of 7.

In addition to the $e_{aq}^{-}$, the *G*-values of oxidizing species, namely the OH• and ${\text{H}}_{2}{\text{O}}_{2}$ were computed and assessed. The results are presented in Figure 2. G(OH•) and $\text{G}\left({\text{H}}_{2}{\text{O}}_{2}\right)$ were simulated at various temperatures ranging from 25 °C to 150 °C. These values were then compared with published experimental data (Elliot and Bartels 2009), as well as simulation results obtained from Monte Carlo track structure codes IONLYS-TRACION and IONLYS-TRACELE (Plante 2011b), and TRACIRT (Hervé du Penhoat et al 2000). The trends observed in the G(OH•) and $\text{G}\left({\text{H}}_{2}{\text{O}}_{2}\right)$ values align closely with the trends reported in the published data. As the temperature increases, $\text{G}\left({\text{H}}_{2}{\text{O}}_{2}\right)$ decreases, which is consistent with the findings reported by Plante (2011b). This behavior can be attributed to the fact that ${\text{H}}_{2}{\text{O}}_{2}$ is predominantly formed through the self-reaction of OH•. The temperature-dependent $\text{G}\left({\text{H}}_{2}{\text{O}}_{2}\right)$ simulated in this study agrees with the outcomes presented by Hervé du Penhoat et al (2000) (Hervé du Penhoat et al 2000). The authors concluded that the reaction rate constant for the self-reaction of OH• decreases with temperature, leading to an increase in G(OH•) and a decrease in $\text{G}\left({\text{H}}_{2}{\text{O}}_{2}\right)$ (Hervé du Penhoat et al 2000). Figure 3 illustrates the time-evolution of *G*-values for reactive species generated at different temperatures ranging from 25 °C to 150 °C, considering an incoming electron energy of 1 MeV. The time interval was limited to 1 μs, as described in section 3.3. The uncertainties associated with $\text{G}\left({\text{H}}^{•}\right)$, $\text{G}\left({\text{H}}_{2}\right)$, $\text{G}\left({\text{H}}_{2}{\text{O}}_{2}\right)$, G(OH•), and $\text{G}\left({\text{OH}}^{-}\right)$ at various temperatures were found to be within 0.31%. In general, as observed in the simulation by Plante (2011) (Plante 2011b), the $\text{G}{(}^{•}\text{OH})$ and $\text{G}\left({\text{H}}^{•}\right)$ decreased over time due to radical recombination, leading to the formation of molecular products. Conversely, the $\text{G}\left({\text{H}}_{2}\right)$ and $\text{G}\left({\text{H}}_{2}{\text{O}}_{2}\right)$ increased as a function of time. As demonstrated in figure 3, the $\text{G}\left({\text{H}}_{2}\right)$ exhibited an increase with temperature. This can be attributed to the fact that H_2_ is primarily generated through the recombination of the $e_{aq}^{-}(e_{aq}^{-}+e_{aq}^{-}\to {\text{H}}_{2}+2{\text{OH}}^{-})$, which is a diffusion-controlled reaction. Consequently, the reaction rate constant for this process increases at a greater rate than the rate at which individual species diffuse out of the spur. As temperature rises, the recombination of radical species within the spurs occurs at a faster rate compared to diffusion, resulting in the production of a greater number of molecular recombination products (Hervé du Penhoat et al 2000). On the other hand, the $\text{G}\left({\text{H}}^{•}\right)$ value did not significantly change as the temperature increased. This can be attributed to four different reactions involved in the generation and decay of ${\text{H}}^{•}$, which include the interactions of $e_{aq}^{-}$ and ${\text{H}}^{+}$ leading to ${\text{H}}^{•}$ formation, ${\text{H}}^{•}$ reacting with OH• to produce ${\text{H}}_{2}\text{O}$, $e_{aq}^{-}$ reacting with ${\text{H}}^{•}$ resulting in ${\text{H}}_{2}$ and ${\text{OH}}^{-}$, and finally, $e_{aq}^{-}$ reacting with ${\text{H}}_{2}\text{O}$ leading to the formation of ${\text{H}}^{•}$ and ${\text{OH}}^{-}$ (Hervé du Penhoat et al 2000).

In this study, material balance tests between the reducing species and oxidizing species were performed to verify the results. *G*-values for $e_{aq}^{-}$, ${\text{H}}_{2}$, OH•, OH•, ${\text{H}}_{2}{\text{O}}_{2}$_,_ and ${\text{HO}}_{2}$ at different temperatures from 25 °C to 150 °C were calculated according to the material balance equations (5), (6) and (7), with a pH value of 7, incoming electron energy of 1 MeV and cut time of 1 μs. As presented in table 1, the material balance tests were satisfied within a 0.44% difference.

### 4.2. Effect of pH values on *G*-values for reactive species

**Figure 4.**
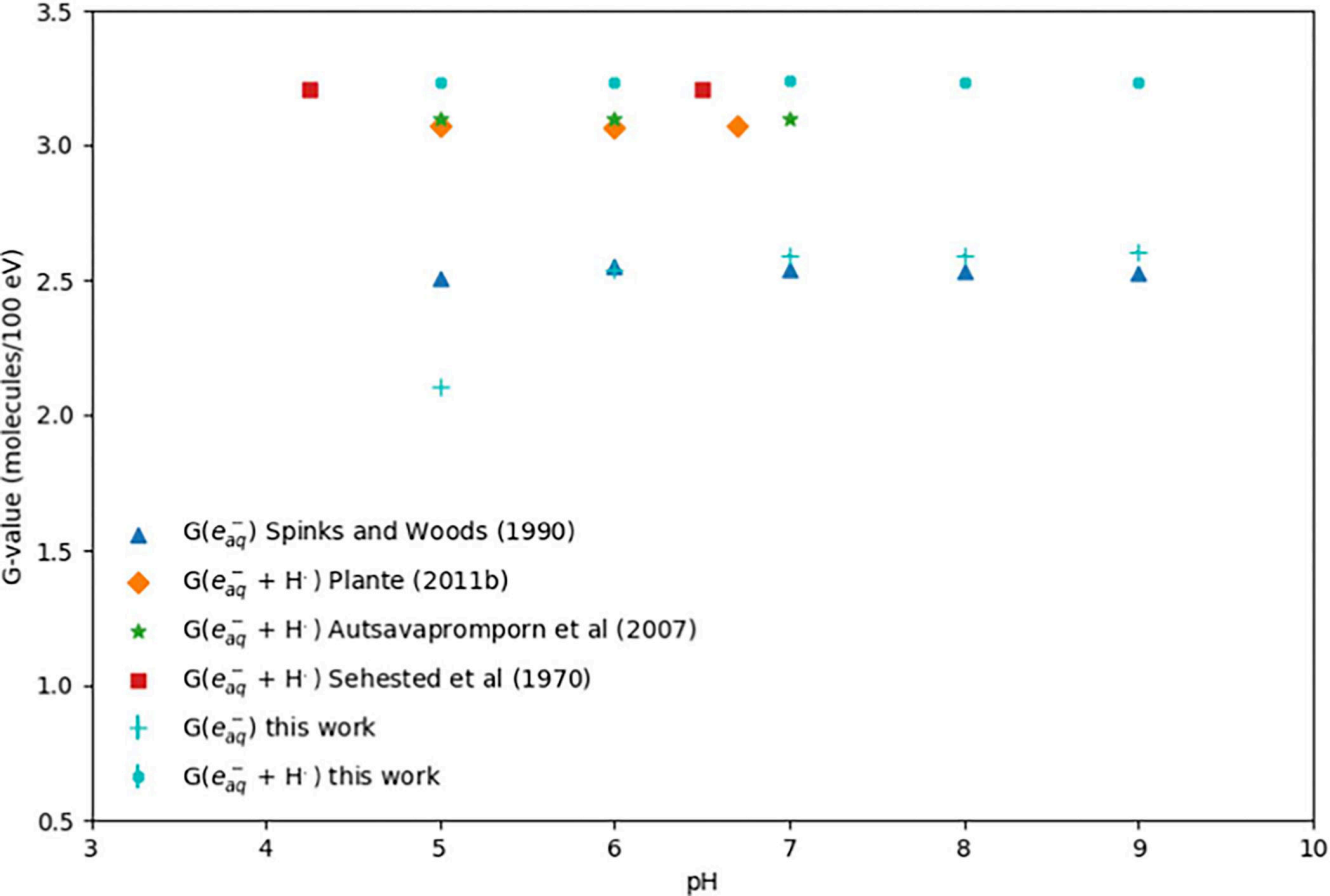
$\text{G}\left(e_{aq}^{-}\right)$ and $\text{G}\left(e_{aq}^{-}+{\text{H}}^{•}\right)$ at different pH values from 5 to 9 simulated in this work and the comparison with published values. The results were compared with the published pulse radiolysis experimental results (Sehested et al 1970, Spinks and Woods 1990) and simulation results from Monte Carlo track structure codes IONLYS-IRT (Autsavapromporn et al 2007), IONLYS-TRACION and IONLYS-TRACELE (Plante 2011b).

Figure 4 shows the $\text{G}\left(e_{aq}^{-}\right)$ and $\text{G}\left(e_{aq}^{-}+{\text{H}}^{•}\right)$ at different pH values from 5 to 9 simulated in this work. The results were compared with the published pulse radiolysis experimental results (Sehested et al 1970, Spinks and Woods 1990) and simulation results from Monte Carlo track structure codes IONLYS-IRT (Autsavapromporn et al 2007), IONLYS-TRACION and IONLYS-TRACELE (Plante 2011b). The $\text{G}\left(e_{aq}^{-}+{\text{H}}^{•}\right)$ was also presented in this Figure to fairly compare with experimental data. The $\text{G}\left(e_{aq}^{-}\right)$ at all simulated pH values were: 2.10 (pH = 5),2.54 (pH = 6), 2.59 (pH = 7), 2.59 (pH = 8) and 2.60 (pH = 9). In this work, the uncertainties of the pH-dependent $\text{G}\left(e_{aq}^{-}\right)$ were less than 0.19%. The trend of the simulated $\text{G}\left(e_{aq}^{-}\right)$ was also in good agreement with the published data. As can be seen in figure 4, our results for all the simulated pH values agreed well with experimental data within 0.52 ± 0.18% to 3.19 ± 0.19% except at a pH of 5 (Sehested et al 1970, Spinks and Woods 1990). For pH of 5, the difference between our simulated result and experimental data from Sprinks and Woods (1990) (Spinks and Woods 1990) was 15.99 ± 0.16%. Overall, considering the additions of solutes in determining the yield of radiolytic species, and thereby difficulties in controlling the pH values (Getoff 1989, 1996), the agreements are acceptable. Regarding simulation results performed with other code packages, our results agreed with the data within 4.40 ± 0.15% to 5.53 ± 0.16% (Autsavapromporn et al 2007, Plante 2011b). In the acid conditions, hydrogen ion (${\text{H}}^{+}$), which can react with $e_{aq}^{-}$ and produce ${\text{H}}^{•}$, are significantly produced, resulting in a low yield of $e_{aq}^{-}$ and a high yield of OH• (Buxton et al 1988, Spinks and Woods 1990, Getoff 1996). In the pH range between 4 and 7, the $\text{G}\left(e_{aq}^{-}+{\text{H}}^{•}\right)$ kept constant and were independent of the pH values. This can be explained by the fact that the main reaction of $e_{aq}^{-}$ in this pH range is with the ${\text{H}}^{+}$, which converts the ${\text{H}}^{+}$ to ${\text{H}}^{•}$ (Autsavapromporn et al 2007). At lower pH values, the reaction rate constant of the $e_{aq}^{-}$ recombination also increases (Marin et al 2007).

**Figure 5.**
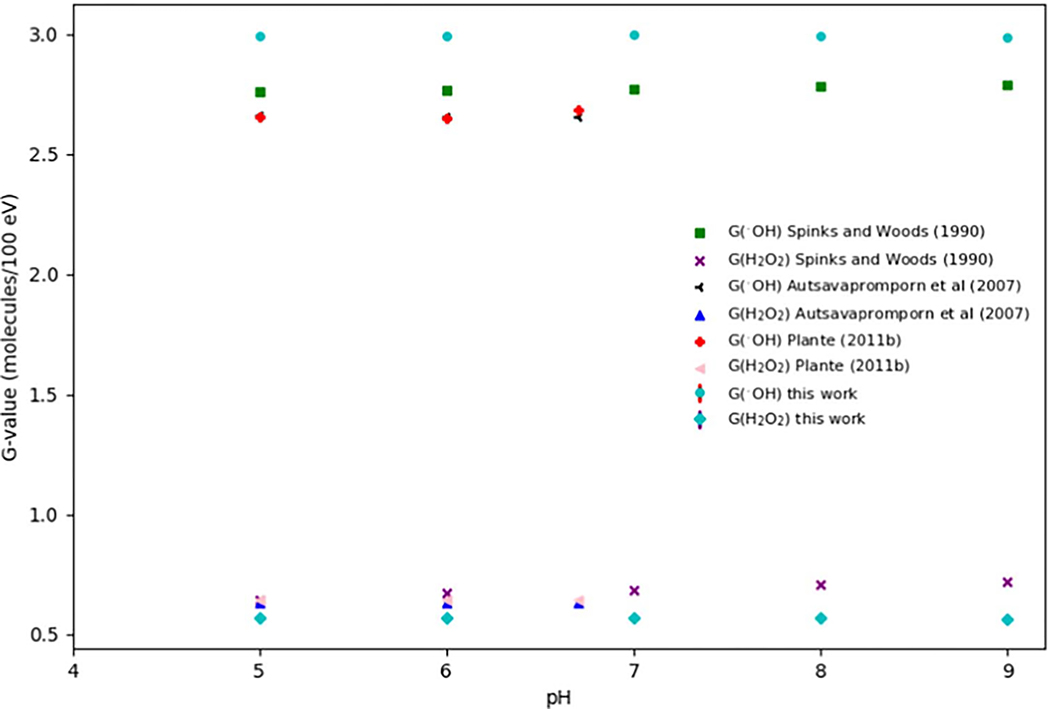
$\text{G}{(}^{•}\text{OH})$ and $\text{G}\left({\text{H}}_{2}{\text{O}}_{2}\right)$ at different pH values from 5 to 9 were simulated in this work and compared with the published work. The results were compared with the published experimental results (Spinks and Woods 1990) and simulation results from Monte Carlo track structure codes IONLYS-IRT (Autsavapromporn et al 2007), IONLYS-TRACION and IONLYS-TRACELE (Plante 2011b).

**Figure 6.**
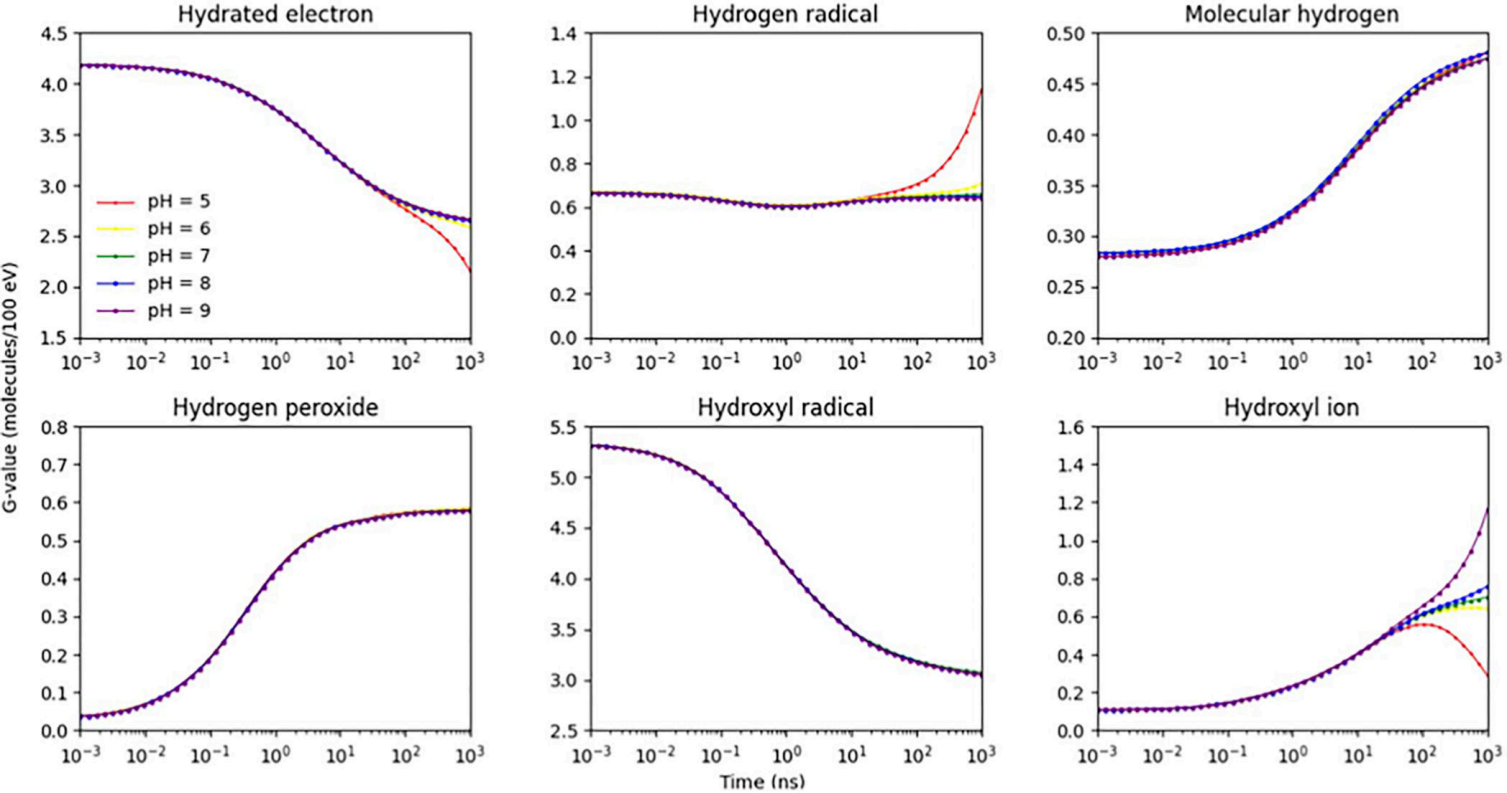
Time-evolution of *G*-values for generated reactive species at different pH values from 5 to 9 with the incoming electron energy of 1 MeV and temperature of 25 °C.

Figure 5 shows *G*-values for oxidizing species $\text{G}{(}^{•}\text{OH})$ and $\text{G}\left({\text{H}}_{2}{\text{O}}_{2}\right)$ at different pH values from 5 to 9 simulated in this work. The results were compared with the published experimental results (Spinks and Woods 1990) and simulation results from Monte Carlo track structure codes IONLYS-IRT (Autsavapromporn et al 2007), IONLYS-TRACION and IONLYS-TRACELE (Plante 2011b). The observed trends in G(OH•) and $\text{G}\left({\text{H}}_{2}{\text{O}}_{2}\right)$ align well with those reported in the published work. The G(OH•) kept constant in the pH range from 4 to 7 and increased with lower pH values. This is because, within the pH range of 4 to 7, the reaction involving $e_{aq}^{-}$ and ${\text{H}}^{+}$ resulting in the formation of ${\text{H}}^{•}$, which is the opposite of scavenging capacity, happens around the same time as the completion of spur expansion. Once the reaction $e_{aq}^{-}+{\text{H}}^{+}\to {\text{H}}^{•}$ takes place, the majority of the initial events in the spur expansion process have already occurred, leading to the generation of most of the reactive species (Plante 2011b). Figure 6 shows the time-evolution of *G*-values for different generated reactive species at different pH values from 5 to 9. Uncertainties for $\text{G}({\text{H}}^{•})$, $\text{G}({\text{H}}_{2})$, $\text{G}({\text{H}}_{2}{\text{O}}_{2})$, G(OH•) and $\text{G}({\text{OH}}^{-})$ at different pH values were within 0.32%.

The material balance equations were also calculated at different pH values. The results are presented in table 2. The material balance tests were satisfied within 0.19% for pH values of 5, 6, 7, 8, and 9.

### 4.3. Impact

In this project, temperature-dependent scaling functions for the chemical parameters were integrated into the GEANT4-DNA. The GEANT4-DNA was updated to automatically change the chemical parameters based on published databases. The users can use the IRT method with different input chemical parameters. At room temperature and neutral pH, the functions converge to the default GEANT4-DNA chemical parameters.

Accurate knowledge of *G*-values under correct temperatures and pH values is important in many fields such as studying the biological damage caused by both conventional (Ramos-Méndez et al 2021) and FLASH radiation (Boscolo et al 2021). In addition, radiolysis of water is important in the field of dosimetry such as water calorimetry (Ross and Klassen 1996) and $e_{aq}^{-}$ dosimetry (Fielden and HART 1968, Mégrourèche 2020) as well as in nuclear reactor technology (McCracken et al 1998, Bartels et al 2013, Kanjana et al 2013). In $e_{aq}^{-}$ dosimetry, accurate determination of $\text{G}\left(e_{aq}^{-}\right)$ is important to obtain an accurate absorbed dose measured with the $e_{aq}^{-}$ dosimeter prototype (Mégrourèche 2020).

### 4.4. Limitations of the work and future study

In this work, changes in temperatures and pH values were considered to be independent events. However, there does exist a dependency between temperatures and pH values. The definition of pH is based on the amount of ${\text{H}}^{+}$ available in the solution. The relationship between pH and ${\text{H}}^{+}$ concentration can be expressed as $\text{pH}=-{\text{log}}_{10}\left[H^{+}\right]/\text{mol}\;{\text{l}}^{-1}$ The self-dissociation activity of water increases with increasing temperature (Sweeton et al 1974, Geissler et al 2001). Moreover, to investigate the effect of pH value on the yields, ionic strength is expected to affect media with high acidity. Those scenarios were not simulated/considered in this work. In future work, the relationship between temperature and pH will be considered and added to the GEANT4-DNA code. Also, further validation using higher LET is required.

## 5. Conclusions

In this work, modifications to the IRT method were successfully added to the GEANT4-DNA source code to simulate *G*-values for reactive species produced in water radiolysis.*G*-values for $e_{aq}^{-}$, ${\text{H}}^{•}$$,{\text{H}}_{2}$, ${\text{H}}_{2}{\text{O}}_{2}$, OH•, and ${\text{OH}}^{-}$ for different physical parameters were obtained and analyzed. Our temperature-dependent results agreed with experimental data within 0.64% to 9.79%, and with simulated data within 3.52% to 12.47%. The pH-dependent results agreed well with experimental data within 0.52% to 3.19% except at a pH of 5 (15.99%) and with simulated data within 4.40% to 5.53%. The uncertainties were below ±0.20%. Overall our results agreed better with experimental than simulation data. The *G*-values for the reactive species simulated were consistent with or could be explained by the conclusions drawn in published work.

## Figures and Tables

**Table 1.** Material balance tests between the primary species at different temperatures from 25 °C to 150 °C, with a pH value of 7, an energy of 1 MeV, and a cut time of 1 μs.

Physical parameter	Material balance
Temperature (°C)	$\left|{\text{G}}_{red}-{\text{G}}_{ox}\right|$

25	0.11%
50	0.21%
75	0.31%
100	0.31%
125	0.24%
150	0.44%

**Table 2.** Material balance tests between the primary species at different pH values from 5 to 9, with a temperature of 25 °C, an incoming electron energy of 1 MeV, and a cut time of 1 μs.

Physical parameter	Material balance
pH values	$\left|{\text{G}}_{red}-{\text{G}}_{ox}\right|$

5	0.19%
6	0.11%
7	0.11%
8	0.07%
9	0.07%

## Data Availability

All data that support the findings of this study are included within the article (and any supplementary information files). Data will be available from 24 March 2023.

## Appendix A. Reaction rate constants

Based on the reaction rate constants, diffusion-controlled reactions may be classified into six types (Elliot 1994, Frongillo et al 1998). Type 1 reactions are totally diffusion-controlled (Table A1), which means that their reaction rates are completely governed by diffusion. Type 2 reactions are partially diffusion controlled (Tables A2, A3, A4, A5), their reaction rates are governed by diffusion but also the reaction rates of reactive loss (loss of reactants). Type 3 reactions are totally diffusion controlled, but their reactants are both ions, so electrical interactions must be considered (Table A6). Type 4 reactions are partially diffusion controlled, but their reactants are also ions (Table A7). Type 5 reactions are totally diffusion controlled, but the molecular spin is considered in the equation. Finally, type 6 reactions are non-diffusion controlled and are mainly first-order or pseudo-first-order reactions (Table A8). Pseudo-first-order reactions are those where one of the reactants has a considerably higher concentration than the other reactants and is considered to be a ‘background molecule’ (Plante 2011b, Plante and Devroye 2017).

**Table A1.** Temperature-dependent polynomials of reaction rate constants of type 1 (totally diffusion controlled) reactions with the gas constant (R) taken as 8.31 J·K^−1^ and temperature t in Kelvin. Values at 25 °C are also indicated. When not specified, polynomial values are from (Elliot and Bartels 2009). Otherwise, the references are: (†)(Elliot 1994), (‡) (Burns and Marsh 1981).

	Type 1 reactions	

Reaction	Polynomial (M^−1^ s^−1^)	Value at 298.15 k (25 °C)

$\text{R}1.1:\text{H}+\text{H}\to {\text{H}}_{2}$	$2.70\times 10^{12}{\text{e}}^{\left(-1867.5/t\right)}$	$0.51\times 10^{10}$
$\text{R}1.2:{\text{e}}_{\text{aq}}^{-}+\text{H}+\left({\text{H}}_{2}\text{O}\right)\to {\text{H}}_{2}+{\text{OH}}^{-}$	$1.14\times 10^{13}{\text{e}}^{-1795.7/t}$	$2.76\times 10^{10}$
R1.3:H+O(3p)→OH	$2.03\times 10^{10}{\text{e}}^{-12.6/(Rt)‡}$	$2.02\times 10^{10}$
$\text{R}1.4:\text{H}+{\text{O}}^{-}\to {\text{OH}}^{-}$	$2.03\times 10^{10}{\text{e}}^{-12.6/(Rt)‡}$	$2.02\times 10^{10}$
$\text{R}1.5:\text{OH}+\text{O}(3\text{p})\to {\text{HO}}_{2}$	$2.03\times 10^{10}{\text{e}}^{-12.6/(Rt)‡}$	$2.02\times 10^{10}$
$\text{R}1.6:{\text{HO}}_{2}+\text{O}(3\text{p})\to {\text{O}}_{2}+\text{OH}$	$2.03\times 10^{10}{\text{e}}^{-12.6/(Rt)‡}$	$2.02\times 10^{10}$
$\text{R}1.7:\text{O}(3\text{p})+\text{O}(3\text{p})\to {\text{O}}_{2}$	$2.21\times 10^{10}{\text{e}}^{-12.6/(Rt)‡}$	$2.20\times 10^{10}$

**Table A2.** Temperature-dependent polynomials of reaction rate constants of type 2 (1/4) (partially diffusion controlled) reactions with the gas constant (R) taken as 8.31 J·K^−1^ and temperature t in Kelvin. Values at 25 °C are also indicated. When not specified, polynomial values are from (Elliot and Bartels 2009). Otherwise, the references are: (†)(Elliot 1994), (‡)(Burns and Marsh 1981).

	Type 2 reactions	

Reaction	Polynomial (M^−1^ s^−1^)	Value at 298.15 k(25 °C)

$\text{R}2.1:\text{OH}+\text{H}\to {\text{H}}_{2}\text{O}$	$4.26\times 10^{11}{\text{e}}^{-1091.9/t}$	$1.09\times 10^{10}$
$\text{R}2.2:\text{H}+{\text{H}}_{2}{\text{O}}_{2}\to \text{OH}+{\text{H}}_{2}\text{O}$	$1.79\times 10^{11}{\text{e}}^{-2533.6/t}$	$3.65\times 10^{7}$
$\text{R}2.3:\text{H}+{\text{OH}}^{-}⇌{\text{e}}_{\text{aq}}^{-}+{\text{H}}_{2}\text{O}$	$\text{logR}2.3=22.970-1.971\times 10^{4}/t+1.137\times 10^{7}/t^{2}-2.991\times 10^{9}/t^{3}+2.803\times 10^{11}/t^{4}$	$2.44\times 10^{7}$
$\text{R}2.4:\text{H}+{\text{O}}_{2}\to {\text{HO}}_{2}$	$\text{LogR}2.4=10.704+2.840\times 10^{2}/t-1.369\times 10^{5}/t^{2}$	$1.31\times 10^{10}$
$\text{R}2.5:\text{H}+{\text{HO}}_{2}\to {\text{H}}_{2}{\text{O}}_{2}$	$5.17\times 10^{12}{\text{e}}^{-1824.2/t}$	$1.13\times 10^{10}$
$\text{R}2.6:\text{H}+{\text{O}}_{2}^{-}\to {\text{HO}}_{2}^{-}$	$5.17\times 10^{12}{\text{e}}^{-1824.2/t}$	$1.13\times 10^{10}$
$\text{R}2.7:\text{OH}+\text{OH}\to {\text{H}}_{2}{\text{O}}_{2}$	$\text{LogR}2.7=8.054+2.193\times 10^{3}/t-7.395\times 10^{5}/t^{2}+6.870\times 10^{7}/t^{3}$	$4.81\times 10^{9}$
$\text{R}2.8:\text{OH}+{\text{H}}_{2}{\text{O}}_{2}\to {\text{HO}}_{2}+{\text{H}}_{2}\text{O}$	$7.68\times 10^{9}{\text{e}}^{-1661.4/t}$	$2.90\times 10^{7}$

**Table A3.** Temperature-dependent polynomials of reaction rate constants of type 2 (2/4) (partially diffusion controlled) reactions with the gas constant (R) taken as 8.31 J·K^−1^ and temperature t in Kelvin. Values at 25 °C are also indicated. When not specified, polynomial values are from (Elliot and Bartels 2009). Otherwise, the references are: (†)(Elliot 1994), (‡)(Burns and Marsh 1981).

	Type 2 reactions	

Reaction	Polynomial(M^−1^ s^−1^)	Valueat298.15 k(25 °C)

$\text{R}2.9:\text{OH}+{\text{H}}_{2}\to \text{H}+{\text{H}}_{2}\text{O}$	$\text{LogR}2.9=-11.556+3.2546\times 10^{4}/t-1.8623\times 10^{7}/t^{2}+4.5543\times 10^{9}/t^{3}-4.1364\times 10^{11}/t^{4}$	$3.95\times 10^{7}$
$\text{R}2.10:{\text{e}}_{aq}^{-}+\text{OH}\to {\text{OH}}^{-}$	$\text{LogR}2.10=13.123-1.023\times 10^{3}/t+7.634\times 10^{4}/t^{2}$	$3.55\times 10^{10}$
$\text{R}2.11:\text{OH}+{\text{OH}}^{-}\to {\text{O}}^{-}+{\text{H}}_{2}\text{O}$	$\text{LogR}2.11=13.339-2.220\times 10^{3}/t+7.333\times 10^{5}/t^{2}-1.065\times 10^{8}/t^{3}$	$1.33\times 10^{10}$
$\text{R}2.12:\text{OH}+{\text{HO}}_{2}\to {\text{O}}_{2}+{\text{H}}_{2}\text{O}$	$1.29\times 10^{11}{\text{e}}^{-799.2/t}$	$8.84\times 10^{9}$
$\text{R}2.13:\text{OH}+{\text{O}}_{2}^{-}\to {\text{O}}_{2}+{\text{OH}}^{-}$	$8.77\times 10^{11}{\text{e}}^{-1306.2/t}$	$1.09\times 10^{10}$
$\text{R}2.14:\text{OH}+{\text{HO}}_{2}^{-}\to {\text{H}}_{2}\text{O}+{\text{O}}_{2}^{-}$	$4.5\times 10^{12}{\text{e}}^{-1877.71360/t}†$	$8.29\times 10^{9}$
$\text{R}2.15:{\text{HO}}_{2}^{-}+{\text{O}}^{-}\to {\text{OH}}^{-}+{\text{O}}_{2}^{-}$	$1.45\times 10^{13}{\text{e}}^{-2928.5/t}$	$7.86\times 10^{8}$
$\text{R}2.16:\text{OH}+{\text{O}}_{3}^{-}\to {\text{O}}_{2}^{-}+{\text{HO}}_{2}$	$8.77\times 10^{11}{\text{e}}^{-1306.2/t}$	$1.09\times 10^{10}$

**Table A4.** Temperature-dependent polynomials of reaction rate constants of type 2 (3/4) (partially diffusion controlled) reactions with the gas constant (R) taken as 8.31 J·K^−1^ and temperature t in Kelvin. Values at 25 °C are also indicated. When not specified, polynomial values are from (Elliot and Bartels 2009). Otherwise, the references are: (†)(Elliot 1994), (‡)(Burns and Marsh 1981).

	Type 2 reactions	
Reaction	Polynomial (M^−1^ s^−1^)	Value at 298.15 k (25 °C)
$\text{R}2.17:{\text{e}}_{aq}^{-}+{\text{H}}_{2}{\text{O}}_{2}\to {\text{OH}}^{-}+\text{OH}$	$7.70\times 10^{12}{\text{e}}^{-1889.6/t}$	$1.36\times 10^{10}$
$\text{R}2{.18:\text{H}}_{2}{\text{O}}_{2}+{\text{OH}}^{-}⇌{\text{HO}}_{2}^{-}+{\text{H}}_{2}\text{O}$	$\text{LogR}2.18=13.339-2.220\times 10^{3}/t+7.333\times 10^{5}/t^{2}-1.065\times 10^{8}/t^{3}$	$1.33\times 10^{10}$
$\text{R}2.19:{\text{H}}_{2}{\text{O}}_{2}+\text{O}(3\text{p})\to {\text{HO}}_{2}+\text{OH}$	$2.99\times 10^{11}{\text{e}}^{-1876.36438/t†}$	$5.53\times 10^{8}$
$\text{R}2{.20:\text{H}}_{2}{\text{O}}_{2}+{\text{O}}^{-}\to {\text{HO}}_{2}+{\text{OH}}^{-}$	$2.99\times 10^{11}{\text{e}}^{-1876.36438/t†}$	$5.53\times 10^{8}$
$\text{R}2{.21:\text{H}}_{2}+\text{O}(3\text{p})\to \text{OH}+\text{H}$	$4.837\times 10^{3}{\text{e}}^{-34.9/(Rt)}‡$	$4.77\times 10^{3}$
$\text{R}2.22:{\text{H}}_{2}+{\text{O}}^{-}\to \text{H}+{\text{OH}}^{-}$	$2.32\times 10^{10}{\text{e}}^{-1550.5/t}$	$1.28\times 10^{8}$
$\text{R}2.23:e_{aq}^{-}+{\text{O}}_{2}\to {\text{O}}_{2}^{-}$	$2.52\times 10^{12}{\text{e}}^{-1401.5/t}$	$2.29\times 10^{10}$
$\text{R}2{.24:\text{e}}_{aq}^{-}+{\text{HO}}_{2}\to {\text{HO}}_{2}^{-}$	$2.46\times 10^{12}{\text{e}}^{-1563.6/t}$	$1.30\times 10^{10}$

**Table A5.** Temperature-dependent polynomials of reaction rate constants of type 2 (4/4) (partially diffusion controlled) reactions with the gas constant (R) taken as 8.31 J·K^−1^ and temperature t in Kelvin. Values at 25 °C are also indicated. When not specified, polynomial values are from (Elliot and Bartels 2009). Otherwise, the references are: (†)(Elliot 1994), (‡)(Burns and Marsh 1981).

	Type 2 reactions	

Reaction	Polynomial (M^−1^ s^−1^)	Value at 298.15 k (25 °C)

$\text{R}2{.25:\text{OH}}^{-}+{\text{HO}}_{2}⇌{\text{O}}_{2}^{-}+{\text{H}}_{2}\text{O}$	$\text{LogR}2.25=13.339-2.220\times 10^{3}/t+7.333\times 10^{5}/t^{2}-1.065\times 10^{8}/t^{3}$	$1.33\times 10^{10}$
$\text{R}2{.26:\text{OH}}^{-}+\text{O}(3\text{p})\to {\text{HO}}_{2}^{-}$	$1.45\times 10^{13}{\text{e}}^{-2928.5/t}$	$7.86\times 10^{8}$
$\text{R}2{.27:\text{O}}_{2}+\text{O}(3\text{p})\to {\text{O}}_{3}$	$3.41\times 10^{11}{\text{e}}^{-1344.9/t}$	$3.74\times 10^{9}$
$\text{R}2{.28:\text{O}}_{2}+{\text{O}}^{-}⇌{\text{O}}_{3}^{-}$	$3.41\times 10^{11}{\text{e}}^{-1344.9/t}$	$3.74\times 10^{9}$
$\text{R}2{.29:\text{HO}}_{2}+{\text{HO}}_{2}\to {\text{H}}_{2}{\text{O}}_{2}+{\text{O}}_{2}$	$2.78\times 10^{9}{\text{e}}^{-2416.4/t}$	$8.40\times 10^{5}$
$\text{R}2{.30:\text{HO}}_{2}+{\text{O}}_{2}^{-}+{\text{H}}_{2}\text{O}\to {\text{H}}_{2}{\text{O}}_{2}+{\text{O}}_{2}+{\text{OH}}^{-}$	$2.63\times 10^{9}{\text{e}}^{-974.3/t}$	$1.00\times 10^{8}$
$\text{R}2{.31:\text{HO}}_{2}+\text{O}(3\text{p})\to {\text{O}}_{2}+\text{OH}$	$2.03\times 10^{10}{\text{e}}^{-12.6/(Rt)‡}$	$2.02\times 10^{10}$

**Table A6.** Temperature-dependent polynomials of reaction rate constants of type 3 (totally diffusion controlled with reactants of ions) reactions with the gas constant (R) taken as 8.31 J·K^−1^ and temperature t in Kelvin. Values at 25 °C are also indicated. When not specified, polynomial values are from (Elliot and Bartels 2009). Otherwise, the references are: (†)(Elliot 1994), (‡)(Burns and Marsh 1981).

	Type 3 reactions	

Reaction	Polynomial (M^−1^ s^−1^)	Value at 298.15 k (25 °C)

$\text{R}3.1:{\text{e}}_{aq}^{-}+{\text{e}}_{aq}^{-}+2{\text{H}}_{2}\text{O}\to {\text{H}}_{2}+2{\text{OH}}^{-}$	$\begin{array}{c} \text{LogR}3.1=12.281-3.786\times 10^{2}/t-6.673\times 10^{4}/t^{2}-1.075\times 10^{7}/t^{3} \end{array}$	$7.16\times 10^{9}$
$\text{R}3.2:{\text{H}}_{3}{\text{O}}^{+}+{\text{OH}}^{-}⇌2{\text{H}}_{2}\text{O}$	$\text{LogR}3.2=20.934-1.236\times 10^{4}/t+6.364\times 10^{6}/t^{2}-1.475\times 10^{9}/t^{3}+1.237\times 10^{1}1/t^{4}$	$1.18\times 10^{11}$
$\text{R}3.3:{\text{H}}_{3}{\text{O}}^{+}+{\text{O}}_{2}^{-}\to {\text{H}}_{2}\text{O}+{\text{HO}}_{2}$	$\text{LogR}3.3=16.410-4.888\times 10^{3}/t+1.622\times 10^{6}/t^{2}-2.004\times 10^{8}/t^{3}$	$5.02\times 10^{10}$

**Table A7.** Temperature-dependent polynomials of reaction rate constants of type 4 (partially diffusion controlled with reactants of ions) reactions with the gas constant (R) taken as 8.31 J·K^−1^ and temperature t in Kelvin. Values at 25 °C are also indicated. When not specified, polynomial values are from (Elliot and Bartels 2009). Otherwise, the references are: (†)(Elliot 1994), (‡)(Burns and Marsh 1981).

	Type 4 reactions	

Reaction	Polynomial (M^−1^ s^−1^)	Value at 298.15 k (25 °C)

$\text{R}4.1:{\text{e}}_{aq}^{-}+{\text{H}}_{3}{\text{O}}^{+}⇌\text{H}+{\text{H}}_{2}\text{O}$	$\text{LogR}4.1=39.127-3.888\times 10^{4}/t+2.054\times 10^{7}/t^{2}-4.899\times 10^{9}/t^{3}+4.376\times 10^{11}/t^{4}$	$2.10\times 10^{10}$
$\text{R}4.2:{\text{e}}_{aq}^{-}+{\text{O}}_{2}^{-}+{\text{H}}_{2}\text{O}\to {\text{H}}_{2}{\text{O}}_{2}+2{\text{OH}}^{-}$	$2.46\times 10^{12}e^{-1563.6/t}$	$1.30\times 10^{10}$
$\text{R}4.3:{\text{e}}_{aq}^{-}+{\text{HO}}_{2}^{-}\to {\text{O}}^{-}+{\text{OH}}^{-}$	$1.75\times 10^{12}e^{-1852.77/t}†$	$3.50\times 10^{9}$
$\text{R}4.4:{\text{e}}_{aq}^{-}+{\text{O}}^{-}\to {\text{OH}}^{-}+{\text{OH}}^{-}$	$5.6\times 10^{11}e^{-951.64/t}†$	$2.31\times 10^{10}$
$\text{R}4.5:{\text{H}}_{3}{\text{O}}^{+}+{\text{O}}_{2}^{-}\to {\text{HO}}_{2}+{\text{H}}_{2}\text{O}$	$\text{LogR}4.5=16.410-4.888\times 10^{3}/t+1.622\times 10^{6}/t^{2}-2.004\times 10^{8}/t^{3}$	$5.02\times 10^{10}$
$\text{R}4.6:{\text{H}}_{3}{\text{O}}^{+}+{\text{HO}}_{2}^{-}\to {\text{H}}_{2}{\text{O}}_{2}+{\text{H}}_{2}\text{O}$	$\text{LogR}4.6=16.410-4.888\times 10^{3}/t+1.622\times 10^{6}/t^{2}-2.004\times 10^{8}/t^{3}$	$5.02\times 10^{10}$
$\text{R}4.7:{\text{H}}_{3}{\text{O}}^{+}+{\text{O}}^{-}\to {\text{OH}}^{-}+{\text{H}}_{2}\text{O}$	$\text{LogR}4.7=16.410-4.888\times 10^{3}/t+1.622\times 10^{6}/t^{2}-2.004\times 10^{8}/t^{3}$	$5.02\times 10^{10}$
$\text{R}4.8:{\text{O}}_{2}^{-}+{\text{O}}^{-}+{\text{H}}_{2}\text{O}\to {\text{O}}_{2}^{-}+{\text{OH}}^{-}+{\text{OH}}^{-}$	$3.41\times 10^{11}{\text{e}}^{-1344.9/t}$	$3.74\times 10^{9}$
$\text{R}4.9:{\text{HO}}_{2}^{-}+{\text{O}}^{-}\to {\text{O}}_{2}^{-}+{\text{OH}}^{-}$	$1.45\times 10^{13}{\text{e}}^{-2928.5/t}$	$7.86\times 10^{8}$
$\text{R}4.10:{\text{O}}^{-}+{\text{O}}^{-}+2{\text{H}}_{2}\text{O}\to {\text{H}}_{2}{\text{O}}_{2}+{\text{OH}}^{-}+{\text{OH}}^{-}$	$2.21\times 10^{10}{\text{e}}^{-12.6/(Rt)‡}$	$2.20\times 10^{10}$
$\text{R}4.11:{\text{O}}^{-}+{\text{O}}_{3}^{-}\to {\text{O}}_{2}^{-}+{\text{O}}_{2}^{-}$	$3.41\times 10^{11}e^{-1344.9/t}‡$	$3.74\times 10^{9}$

**Table A8.** Temperature-dependent polynomials of reaction rate constants of type 6 (non-diffusion controlled) reactions with the gas constant (R) taken as 8.31 J·K^−1^ and temperature t in Kelvin. Values at 25 °C are also indicated. When not specified, polynomial values are from (Elliot and Bartels 2009). The K values, expressed in Molar units, are taken from (Elliot and Bartels 2009). Otherwise, the references are (†)(Elliot 1994), (‡)(Burns and Marsh 1981). (B) means the background species. [${\text{H}}_{2}\text{O}$] is presented in table C1.

	Type 6 Reactions	

Reaction	Polynomial (s^−1^)	Value at 298.15 k (25 °C)

$\text{R}6.1:{\text{O}}_{3}^{-}+{\text{O}}^{-}\to 2{\text{O}}_{2}^{-}$	$3.20\times 10^{11}{\text{e}}^{-5552.1/t}$	$2.6\times 10^{3}$
$\text{R}6{.2:\text{HO}}_{2}+{\text{H}}_{2}\text{O}\to {\text{H}}_{3}^{+}+{\text{O}}_{2}^{-}$	$\text{R}4.5•{\text{K}}_{HO_{2}}•\left[{\text{H}}_{2}\text{O}\right]$	$4.28\times 10^{7}$
$\text{R}6.3:\text{H}+{\text{H}}_{2}\text{O}\to {\text{e}}_{aq}^{-}+{\text{H}}_{3}{\text{O}}^{+}$	$\text{R}4.1•{\text{K}}_{H}•\left[{\text{H}}_{2}\text{O}\right]$	$3.23\times 10^{2}$
$\text{R}6.4:{\text{e}}_{aq}^{-}+{\text{H}}_{2}\text{O}\to \text{H}+{\text{OH}}^{-}$	$\frac{R2•3•K_{{\text{H}}_{2}\text{O}}}{K_{\text{H}}}•\left[{\text{H}}_{2}\text{O}\right]$	$8.73\times 10^{2}$
$\text{R}6.5:{\text{O}}_{2}^{-}+{\text{H}}_{2}\text{O}\to {\text{HO}}_{2}+{\text{OH}}^{-}$	$2.266\times 10^{10}{\text{e}}^{-2897.95134/t}•\left[{\text{H}}_{2}\text{O}\right]†$	$7.54\times 10^{7}$
$\text{R}6.6:{\text{HO}}_{2}^{-}+{\text{H}}_{2}\text{O}\to {\text{H}}_{2}{\text{O}}_{2}+{\text{OH}}^{-}$	$\frac{R2.18•K_{{\text{H}}_{2}\text{O}}}{K_{{\text{H}}_{2}{\text{O}}_{2}}}•\left[{\text{H}}_{2}\text{O}\right]$	$7.05\times 10^{7}$
$\text{R}6.7:\text{O}(3\text{p})+{\text{H}}_{2}\text{O}\to \text{OH}+{\text{OH}}^{-}$	$18.4048\times 10^{-41.4/(Rt)}‡$	$1.00\times 10^{3}$
$\text{R}6{.8:\text{O}}^{-}+{\text{H}}_{2}\text{O}\to \text{OH}+{\text{OH}}^{-}$	$\frac{R2.18•K_{{\text{H}}_{2}\text{O}}}{{\text{K}}_{{\text{H}}_{2}{\text{O}}_{2}}}•\left[{\text{H}}_{2}\text{O}\right]$	$7.05\times 10^{7}$
$\text{R}6.9:{\text{e}}_{aq}^{-}+{\text{H}}_{3}{\text{O}}^{+}(\text{B})\to \text{H}+{\text{H}}_{2}\text{O}$	$\text{R}4.1•\left[{\text{H}}_{3}{\text{O}}^{+}(\text{B})\right]$	$2.07\times 10^{3}$
$\text{R}6.10:{\text{O}}_{2}^{-}+{\text{H}}_{3}{\text{O}}^{+}(\text{B})\to {\text{HO}}_{2}+{\text{H}}_{2}\text{O}$	$\text{R}4.5•\left[{\text{H}}_{3}{\text{O}}^{+}(\text{B})\right]$	$4.97\times 10^{3}$
$\text{R}6.11:{\text{OH}}^{-}+{\text{H}}_{3}{\text{O}}^{+}(\text{B})\to 2{\text{H}}_{2}\text{O}$	$\text{R}3.2•\left[{\text{H}}_{3}{\text{O}}^{+}(\text{B})\right]$	$1.16\times 10^{4}$
$\text{R}6.12:{\text{H}}_{3}{\text{O}}^{+}+{\text{OH}}^{-}(\text{B})\to 2{\text{H}}_{2}\text{O}$	$\text{R}3.2•\left[{\text{OH}}^{-}(\text{B})\right]$	$1.16\times 10^{4}$
$\text{R}6{.13:\text{HO}}_{2}^{-}+{\text{H}}_{3}{\text{O}}^{+}(\text{B})\to {\text{H}}_{2}{\text{O}}_{2}+{\text{H}}_{2}\text{O}$	$\text{R}4.6•\left[{\text{H}}_{3}{\text{O}}^{+}(\text{B})\right]$	$4.97\times 10^{3}$
$\text{R}6.14:{\text{O}}^{-}+{\text{H}}_{3}{\text{O}}^{+}(\text{B})\to \text{OH}+{\text{H}}_{2}\text{O}$	$\text{R}4.7•\left[{\text{H}}_{3}{\text{O}}^{+}(\text{B})\right]$	$4.97\times 10^{3}$
$\text{R}6.15:{\text{O}}_{3}^{-}+{\text{H}}_{3}{\text{O}}^{+}(\text{B})\to \text{OH}+{\text{O}}_{2}+{\text{H}}_{2}\text{O}$	$\text{R}3.3•\left[{\text{H}}_{3}{\text{O}}^{+}(\text{B})\right]$	$4.97\times 10^{3}$
$\text{R}6.16:\text{H}+{\text{OH}}^{-}(\text{B})\to {\text{H}}_{2}\text{O}+{\text{e}}_{aq}^{-}$	$\text{R}2.3•\left[{\text{OH}}^{-}(\text{B})\right]$	2.41
$\text{R}6.17:\text{OH}+{\text{OH}}^{-}(\text{B})\to {\text{O}}^{-}+{\text{H}}_{2}\text{O}$	$\text{R}2.11•\left[{\text{OH}}^{-}(\text{B})\right]$	$1.32\times 10^{3}$
$\text{R}6{.18:\text{H}}_{2}{\text{O}}_{2}+{\text{OH}}^{-}(\text{B})\to {\text{HO}}_{2}^{-}+{\text{H}}_{2}\text{O}$	$\text{R}2.18•\left[{\text{OH}}^{-}(\text{B})\right]$	$1.32\times 10^{3}$	$1.32\times 10^{3}$
$\text{R}6.19:{\text{HO}}_{2}+{\text{OH}}^{-}(\text{B})\to {\text{O}}_{2}^{-}+{\text{H}}_{2}\text{O}$	$\text{R}2.25•\left[{\text{OH}}^{-}(\text{B})\right]$	$1.32\times 10^{3}$
$\text{R}6.20:\text{O}+{\text{OH}}^{-}(\text{B})\to {\text{HO}}_{2}^{-}$	$\text{R}2.26•\left[{\text{OH}}^{-}(\text{B})\right]$	$7.78\times 10^{1}$

## Appendix B. Diffusion coefficients

**Table B1.** Diffusion coefficients (1/2) in units of m^2^ s^−1^ with ρ=density, t in Kelvin, and T in Celsius. When not specified, polynomials are from (Kallikragas et al 2014). Otherwise, the references are (†)(Elliot 1994), (⋆)(Schmidt et al 1995).

	Diffusion Coefficient

D	Polynomial(m^2^s^−1^)
${\text{D}}_{OH}$	$(0.00408•{\text{t}}^{1.38897}+\text{p}•\left(1.0/{\text{t}}^{2}-174613.7394/\text{t}+847.03131-0.71041\text{t}\right)+\;{\text{p}}^{2}•\text{log}(\text{p})•\left(1.0/{\text{t}}^{2}-15364.1999/\text{t}+782.41564-0.64852\text{t}\right)+\;{\text{p}}^{2}•\left(1.0/{\text{t}}^{2}+163191.6423/\text{t}-795.71944+0.634849\text{t}\right)1{\text{e}}^{-9}$
${\text{D}}_{O_{2}}$	$1.82779•{\text{t}}^{0.422868}+\text{p}•\left(1.0/{\text{t}}^{2}-102443/\text{t}+334.021-0.119239\text{t}\right)+\;{\text{p}}^{2}•\text{log}(\text{p})•\left(1.0/{\text{t}}^{2}-102959/\text{t}+334.195-0.117517\text{t}\right)+\;{\text{p}}^{2}•\left(1.0/{\text{t}}^{2}+100433/\text{t}-347.059+0.125558\text{t}\right)•1{\text{e}}^{-9}$
${\text{D}}_{H_{2}}$	$116.211•{\text{t}}^{0.0538917}+\text{p}•\left(1.0/{\text{t}}^{2}+372312/\text{t}-1794.21+1.42193\text{t}\right)+\;{\text{p}}^{2}•\text{log}(\text{p})•\left(1.0/{\text{t}}^{2}+476427/\text{t}-1880.99+1.6233•\text{t}\right)+\;{\text{p}}^{2}•\left(1.0/{\text{t}}^{2}-377905/\text{t}+1654.15-1.39764\text{t}\right)•1{\text{e}}^{-9}$
${\text{D}}_{O_{2}^{-}}$	$1.82779•{\text{t}}^{0.42268}+\text{p}•\left(1.0/{\text{t}}^{2}-102443/\text{t}+334.021-0.119239\text{t}\right)+\;{\text{p}}^{2}•\text{log}(\text{p})•\left(1.0/{\text{t}}^{2}-102959/\text{t}+334.195-0.117517•\text{t}\right)+\;{\text{p}}^{2}•\left(1.0/{\text{t}}^{2}+100433/\text{t}-347.059+0.125558•\text{t}\right)-0.55152409-0.25)•{\text{le}}^{-9}={\text{D}}_{{\text{O}}_{2}}\text{scaled}$
${\text{D}}_{O_{3}^{-}}$	$((1.82779•{\text{t}}^{0.42268}+\text{p}•\left(1.0/{\text{t}}^{2}-102443/\text{t}+334.021-0.119239\text{t}\right)+\;{\text{p}}^{2}•\text{log}(\text{p})•\left(1.0/{\text{t}}^{2}-102959/\text{t}+334.195-0.117517\text{t}\right)+\;{\text{p}}^{2}•\left(1.0/{\text{t}}^{2}+100433/\text{t}-347.059+0.125558\text{t}\right)-0.55152409)•1{\text{e}}^{-9}={\text{D}}_{{\text{O}}_{2}}\text{scaled}$
${\text{D}}_{O_{3}}$	${\text{D}}_{O_{3}}={\text{D}}_{O_{3}^{-}}$
${\text{D}}_{O(3p)}$	${\text{D}}_{O(3p)}={\text{D}}_{O_{3}^{-}}$
${\text{D}}_{O^{-}}$	${\text{D}}_{O^{-}}={\text{D}}_{O_{3}^{-}}$

**Table B2.** Diffusion coefficients (2/2) in units of m^2^s^−1^ with ρ=density, t in Kelvin, and T in Celsius. When not specified, polynomials are from (Kallikragas et al 2014). Otherwise, the references are (†) (Elliot 1994), (⋆)(Schmidt et al 1995).

	Diffusion Coefficient

D	Polynomial(m^2^s^−1^)
${\text{D}}_{H}^{+†}$	$5.361\times 10^{-9}+1.659\times 10^{-10}\text{T}-7.48\times 10^{-14}T^{2}-1.0018\times 10^{-16}T^{3}$
${{\text{D}}_{H_{2}O_{2}}}^{†}$	$(0.0046471•t^{1.2939}+\text{p}•\left(1.05646\times 10^{8}/t^{2}-511648/t+691.903-0.1729\text{t}\right)+\;{\text{p}}^{2}•\text{log}(\text{p})•\left(8.88978\times 10^{7}/t^{2}-358381/\text{t}+334.773+0.0213276\text{t}\right)+\;{\text{p}}^{2}•\left(-1.01281\times 10^{8}/t^{2}+475755/\text{t}-608.627+0.116203\text{t}\right))•1{\text{e}}^{-9}$
${\text{D}}_{HO^{-†}}$	$2.666\times 10^{-9}+9.769\times 10^{-11}\text{T}+3.303\times 10^{-13}T^{2}-7.295\times 10^{-16}T^{3}$
${{\text{D}}_{H}}^{†}$	$\begin{array}{c} 5.361\times 10^{-9}+1.659\times 10^{-10}\text{T}-7.48\times 10^{-14}T^{2}-1.0018\times 10^{-16}T^{3}\;-2.46|\times 10^{-09}={\text{D}}_{H^{+}}\text{scaled} \end{array}$
${{\text{D}}_{HO_{2}}}^{†}$	${\text{D}}_{HO_{2}}={\text{D}}_{H_{2}O_{2}}$
${\text{D}}_{HO_{2}^{-†}}$	$(0.0046471•{\text{t}}^{1.2939}+\text{p}•\left(1.05646\times 10^{8}/t^{2}-511648/\text{t}+691.903-0.1729\text{t}\right)+\;{\text{p}}^{2}•\text{log}(\text{p})•\left(8.88978\times 10^{7}/t^{2}-358381/\text{t}+334.773+0.0213276\text{t}\right)+{\text{p}}^{2}•(-1.01281\times 10^{8}/t^{2}+\;475755/\text{t}-608.627+0.116203\text{t})-1.034362355)•1{\text{e}}^{-9}$
${\text{D}}_{e_{aq}^{-}⋆}$	$10^{-0.7638-1058.18/T{\left(254.92/T\right)}^{40}}{\text{e}}^{-4}$

## Appendix C. Water properties

**Table C1.** Water property from Elliot and Bartels (2009) with temperature t in Celsius.

	Water Properties

Water Property	Polynomial
[${\text{H}}_{2}\text{O}$](molel^–1^)	$55.50+6.075\times 10^{-3}\text{t}-4.110\times 10^{-4}t^{2}+1.496\times 10^{-6}{\text{t}}^{3}-2.619\times 10^{-9}{\text{t}}^{4}$
